# The Management of Thyroid Eye Disease: From Current Practice to Future Perspectives

**DOI:** 10.7759/cureus.86483

**Published:** 2025-06-21

**Authors:** Navnit Kaur Dhaliwal, Lubna Razzaq

**Affiliations:** 1 Medicine and Surgery, Queen Mary University of London, London, GBR; 2 Opthalmology, Guy's and St. Thomas' NHS Foundation Trust, London, GBR

**Keywords:** endocrinology and diabetes, graves opthalmology, opthalmology ocular pathology, thyroid eye disease (ted), thyroid peroxidase antibodies

## Abstract

Thyroid eye disease (TED) is a complex autoimmune disease, which can have debilitating and possibly sight-threatening consequences. TED can present with diplopia, strabismus, pain and exophthalmos. Despite pathophysiology not being fully elucidated, key components of the TED pathogenesis pathway have been established. Current management focuses on reducing inflammation in the active phase of TED and subsequently employs surgical interventions to rectify the clinical sequelae that arise post-inflammation. However, given the side effect profiles and varying efficacy of current treatment options, there is a need for newer advanced therapeutic options. Future therapies are focusing on biologic use and small-molecule antagonists. This could revolutionise the treatment of TED, as these therapeutic techniques target specific parts of the TED pathophysiological pathway unlike most of the current treatments available today. The current and future landscape of TED treatment is a rapidly changing scene. This article will provide an insight into the current therapeutic options for TED, whilst discussing future potential treatments.

## Introduction and background

Thyroid eye disease (TED) is an autoimmune condition [1] that affects the orbit of the eye. It is a disfiguring and debilitating condition that has the potential to cause devastating sight-threatening consequences [1]. The prevalence and incidence of TED vary across different regions in the world and between genders [2-4]. The European Group on Graves’ Orbitopathy (EUGOGO) estimates the prevalence of TED to be between 90 to 155 per 100,000 across Europe [1]. Whilst it has been acknowledged in the literature that regional variation exists in TED, the data is limited [1-4]. It has been estimated that in the United States, the prevalence of TED is approximately 250 per 100,000 [1-4], and in Asia, the prevalence ranges between 100 and 300 per 100,000. In terms of gender variation of TED women are more affected than men [2]. The annual incidence rate for TED in women is 16 per 100,000 compared to 2.9 per 100,000 cases in men [2].

TED is mostly associated with hyperthyroidism, but it can also be associated with euthyroidism (normal thyroid function) or hypothyroidism, but this is much less common. In the majority of cases, TED is associated with Graves' disease [1]. Graves' disease is an autoimmune disease, whereby antibodies target and hyperstimulate the thyroid-stimulating hormone receptor (TSHR) on the thyroid gland. A recent meta-analysis, consisting of 57 studies and 26,804 patients, concluded that the overall prevalence of TED in patients with Graves' disease was 40% [1] with varying degrees of severity [1]. Eighty-five percent of cases presented within 18 months before or after a Graves’ disease diagnosis [1]. In light of this, this article will endeavour to discuss our understanding of both current and prospective treatments for TED.

## Review

Risk factors

The onset of TED can be attributed to an intricate interplay between unmodifiable and modifiable risk factors [1]. The single biggest modifiable risk factor contributing to TED is smoking [5]. Patients who are active smokers have more severe manifestations of TED that are often less responsive to immunosuppressive treatment, compared with non-smokers [1]. This highlights the importance of encouraging smoking cessation. Thyroid status is another important modifiable risk. Hyperthyroid or hypothyroid status can contribute to the occurrence of TED; thus, it is vital to maintain euthyroid status [5]. Other modifiable risk factors include selenium deficiency, occurrence of stressful life events, intestinal microbiota composition and thyroid hormone levels [5]. There is also an associated increase of developing TED in patients who require radioactive iodine to treat Graves' disease [5]. Non-modifiable risk factors include female gender, hypercholesterolemia and genetic predisposition. The TSHR auto-antibody (TRAb) level is another important risk factor to consider [5]. The higher the level of TRAb at the point of diagnosis of Graves' disease, the more likely a patient is to develop TED. Furthermore, higher TRAb titres correlate with higher TED severity and activity [5]. It is of note that TRAb concentration can be reduced with anti-thyroid medical treatment or thyroidectomy [5].

Clinical manifestations

Clinical manifestations of TED can vary widely between patients. The most common clinical findings include dry eyes, eyelid retraction, diplopia, strabismus, globe exposure, extraocular muscle inflammation and subsequent enlargement, and retro-orbital fat expansion [6]. The latter three processes lead to exophthalmos and restricted ocular movements. Exophthalmos and eyelid retraction can precipitate ulceration and corneal exposure, which could predispose patients to vision loss [6]. More severe clinical features include periorbital oedema and corneal breakdown. In extreme cases, the increased pressure in the orbital space can cause optic nerve compression, which could lead to irreversible vision loss [6].

Diagnosis

To obtain an accurate diagnosis, a number of different investigations are undertaken such as visual acuity assessment, peripheral vision assessment, slit lamp examination, exophthalmometry, and orthoptic assessment [7]. Formal ophthalmic assessments involve categorising the TED in terms of activity and severity. The EUGOGO scale classifies the severity as mild, moderate to severe and sight-threatening (Table 1 [7]). Although MRI and CT scans are not routinely used to classify severity, they can provide additional information if required [8].

**Table 1:** The severity classification Based on the EUGOGO guidelines [7], the clinical activity of TED is assessed using a clinical activity score (CAS). The CAS consists of seven items, and if a patient presents with more than three of these items, TED is considered to be clinically active, indicating the presence of ongoing inflammation [8]. Such items include assessing for redness of the eyelid and conjunctiva, swelling of the plica, eyelid or conjunctiva, retrobulbar pain or pain on attempted downward or upward gaze and swelling [8]. A 10-point CAS score, which takes into account three additional items, may also be used to evaluate recent changes in CAS activity [8]. GO: Graves’ orbitopathy; TED: thyroid eye disease; EUGOGO: European Group on Graves’ Orbitopathy

Classification	Features
Mild GO	Patients present with one or more of the following [7]: Soft-tissue involvement; lid retraction of less than 2 mm; lack of or intermittent diplopia; corneal exposure that can be treated with lubricants; exophthalmosless than 3 mm above normal for race and gender
Moderate to severe GO	Patients present with two or more of the following [7]: moderate or severe soft-tissue involvement; presence of diplopia; lid retraction more than or equal to 2 mm; exophthalmos more than or equal to 3 mm above normal for race and gender
Sight-threatening GO	Patients present with corneal breakdown; optic neuropathy [7]

Pathophysiology

Substantial advancements have been made in deciphering the pathophysiology of TED [9]. However, the exact mechanism still remains unclear. Orbital fibroblast cells are a key cell type that significantly contributes to TED pathogenesis. Research has highlighted that Graves' disease patients possess an altered form of orbital fibroblasts, compared with healthy controls [10]. In TED, the orbital fibroblasts exhibit increased expression of the TSHR and the insulin-like growth factor-1 receptor (IGF-1R) [9]. As previously mentioned, Graves' disease patients have higher levels of circulating TRAbs. When the TRAbs bind to the TSHR on the orbital fibroblasts, it promotes TSHR and IGF-1R communication. The TSHR/IGF-1R interaction promotes upregulation of signalling pathways that encourage orbital fibroblast differentiation into myofibroblasts and adipocytes, which promotes fibrosis and adipogenesis of the orbit [9].

Given that TED is an autoimmune disease, it is vital to discuss the contribution of immune cells to TED pathophysiology. T cells and B cells are key immune cells that increase inflammation and promote TED development. B cells mainly secrete cytokines such as IFN-γ, TGF-β,IL-4, IL-6 and IL-10, as well as producing increased auto-antibodies [9,10]. These cytokines promote migration of even more T cells and B cells, and other immune cells such as mast cells to migrate into the orbit. This in turn perpetuates the cycle of increased inflammation [9,10]. Furthermore, orbital fibroblasts in TED become hyper-responsive to pro-inflammatory cytokines. These pro-inflammatory cytokines promote the orbital fibroblasts to undergo active cellular remodelling and proliferation, which leads to increased expansion of cellular tissue in the bony orbit [9,10].

Aside from orbital fibroblasts, other cell types, such as orbital fibrocytes and adipocytes, have been found to contribute to TED pathogenesis. Fibrocytes possess the ability to express various receptors such as CD34 receptors [10]. When fibrocytes have the CD34 receptor, they are known as CD34+ fibrocytes. CD34+ fibrocytes express higher levels of the TSHR than CD34- fibrocytes, and when activated can produce high levels of pro-inflammatory cytokines and can differentiate to adipocytes or myofibroblasts, which serve to promote inflammation and increased tissue expansion within the bony orbit [10]. In TED, orbital adipocytes express increased TSHR; this combined with increased TRAb binding promotes increased production of hyaluronic acid and adipogenesis within the orbital cavity [11]. Ultimately, the cycle of increased inflammation and tissue expansion and fibrosis within the orbital cavity gives rise to the clinical manifestation of TED [9].

The natural history of TED follows three distinct phases, as proposed by Rundle and Wilson [1,12]. The first phase is the initial inflammatory active phase, followed by stable or plateau phase and finally an inactive phase. During the inactive phase, inflammation subsides, giving rise to the irregular, misshapen, and enlarged orbital tissue, which give rise to the clinical symptoms of TED.

Current management of TED

TED management depends on whether the disease is inactive or active and how severe the clinical manifestations are. If the patient presents in the active phase, then treatment will focus on suppressing inflammation [7,8]. In the chronic or inactive phase of the TED, management will focus on attempting to restore pre-TED ocular appearance and function [8]. In the active phase, management is mostly medical and in the inactive phase management is mostly surgical [7,8]. Table 2 summarises the possible management options for TED and Table 3 highlights treatment goals with each intervention.

**Table 2:** TED treatment options Potential treatment options that can be utilised during the active or inactive stage. Treatment options include both surgical and medical management. The use of lubricants and encouragement of lifestyle measures should be offered to all patients with TED [7,8]. The treatment recommendations are based on the combination of EUGOGO and American Thyroid Association guidelines [7,8]. TED: Thyroid eye disease

TED Stage	Possible treatments in the active stage	Possible treatment options in the inactive stage
Mild to moderate	6-month selenium supplements, oral glucocorticoids	Orbital decompression, correction of eyelid retraction, blepharoplasties
Moderate to severe	Intravenous glucocorticoids (methylprednisolone), oral mycophenolate, cyclosporine, azathioprine, orbital radiotherapy, teprotumumab, rituximab, tocilizumab, intravenous immunoglobulin	Orbital decompression, blepharoplasties, strabismus surgery, permanent prisms, eyelid correction
Sight threatening	Intravenous glucocorticoids (methylprednisolone), orbital radiotherapy +/- intravenous immunoglobulin	Orbital decompression, lid retraction correction, lubricants and topical antibiotics, tarsorrhaphy

**Table 3:** Treatment goals of current TED management options The table summarises the mechanism of action and treatment goals for the current available treatments for TED [7,8]. TED: Thyroid eye disease

Mode of treatment	Treatment goals
Selenium	Reduces the severity of TED progression
Lubricant eye drops	Aims to prevent or treat corneal exposure; can be used to prevent dry eye syndrome secondary to TED
Glucocorticoids	Reduces immune cell activity/ Reduction in inflammation
Oral mycophenolate	Reduces antibody production in B cells, induces T cell apoptosis, and reduces recruitment of these immune cells into the orbit.
Cyclosporine	Reduces T cell proliferation and interleukin secretion specifically IL-2 secretion
Azathioprine	Reduces immune cell (particularly B and T cells) activity and reduces inflammation
Orbital Radiotherapy	Aims to reduce inflammation in the orbit, promotes inactivation of thyroid eye disease and improves eye motility.
Teprotumumab	This monoclonal antibody inhibits the activity of the IGF-1R by promoting internalisation and degradation of IGF-1R.
Rituximab	Rituximab targets and blocks CD20 surface antigen expressed on B cells thus reducing inflammation in the orbit via B-cell depletion
Tocilizumab	It targets and blocks the activity of IL-6, which is a cytokine known to activate B and T cells, which contributes to a proinflammatory state and adipocyte volume expansion.
Intravenous immunoglobulin	Promotes inactivation of thyroid eye disease, improves diplopia, improves eye motility, utilised in the management of optic nerve decompression.
Orbital decompression	The aim of this surgery is to reduce exapthalmos by removing parts of the orbital wall and/or additional fatty tissue.
Blepharoplasties	Removal of excess skin and fat bulges for aesthetic improvement
Strabismus surgery	Restoration of single vision gaze
Permanent prisms	Restoration of single vision gaze
Tarsorrhaphy	Promotes protection of the corneal surface

Deciding which treatment options to utilise is based on a combination of different factors such as treatment goals, quality of life, availability and cost of treatment, duration of TED, patient age, comorbidities and preferences [7,8]. As such, effective discussions must take place to ensure the patient understands the risks and benefits of each therapeutic option [7,8]. It is imperative to inform patients that predicting the initial response to therapy is challenging [7,8] and they may require multiple rounds of medical treatment and/or additional surgery.

Glucocorticoids

The first-line treatment for TED is usually glucocorticoids [8]. Various studies and trial reports [13-17] have estimated that response rates for intravenous glucocorticoids were around 80% and those for oral glucocorticoids were around 50-60%. Therefore, glucocorticoids are most commonly combined with other therapeutic options and are rarely given as a stand-alone treatment in moderate to severe forms of TED [13,14]. Furthermore, large scale meta-analyses have highlighted that whilst intravenous glucocorticoids can reduce the CAS, they have very limited or no improvement on diplopia and proptosis [13,14,18]. Glucocorticoids can potentially cause a range of side effects, which can include liver failure and death [19-21]. The EUGOGO guidelines recommended doses not exceeding 7.5g per cycle [8], even in the most severe forms of TED to mitigate the potential side effects of glucocorticoids. Glucocorticoids have many benefits namely its cost-effectiveness and improvement of quality of life [13]. However, they provide little benefit in improving diplopia and almost no improvement in proptosis and therefore are rarely used as a stand-alone treatment [13].

Mycophenolate Mofetil (MMF)

Non-steroidal agents such as MMF have been tested either as a stand-alone treatment or in conjunction with glucocorticoids. A randomised clinical trial found that MMF had greater response (91·3% vs 67·9%, P = 0·000) and greater CAS reduction (92·5% vs 70·5%, P < 0·05) than glucocorticoid therapy [22]. Furthermore, the MMF group did not show any signs of disease reactivation, whereas TED reactivation was seen in 7% of the glucocorticoid group [22]. One patient in the glucocorticoid group experienced an adverse effect but none experienced such an effect in the MMF group [22]. However, the study has since been retracted due to a significant error with the data used for analysis. Thus, any results and conclusions drawn from this study are unreliable and invalid [22]. In another randomised, observer-masked, multicentre trial [23] that compared MMF with and without glucocorticoids, benefits were observed only in post hoc analysis in the MMF and glucocorticoid combination group [23]. Despite the limited data, EUGOGO guidelines currently state that MMF can be used for moderate to severe TED [8].

Cyclosporine A and Azathioprine

The data for the use of cyclopsorine A use in TED is limited. Results from randomised control trials and studies suggest that cyclosporine A and glucocorticoids combination had a higher response rate than glucocorticoids or cyclosporine A alone; however, as a single therapy, glucocorticoid treatment fared better [24,25]. Furthermore, cyclosporine A has significant side effects that can severely affect the neuronal, cardiovascular and renal systems [13].

The role of azathioprine in the management in TED is currently unclear. Initial findings of the combined immunosuppression and radiotherapy in thyroid eye disease (CIRTED) trial [26], which was a double-blind, randomised controlled trial suggesting that azathioprine may be beneficial in reducing inflammation post 24 weeks use [26]. However, newer research from the three-year follow up of the CIRTED trial concluded that there is no clear benefit in the addition of azathioprine to glucocorticoids [27]. Furthermore, the findings highlighted that there was no benefit of patients receiving azathioprine compared to placebo in terms of the CAS score [27].

Radiotherapy

Several studies have shown external beam radiation therapy to be effective in reducing CAS, improving ocular motility and diplopia [28-30]. Findings from a recent systematic review [31] highlighted that the side effects associated with radiotherapy for TED are generally minor and manageable, such as dry eye or cataract. Results from other studies and reports highlight that risk of major side effects such as radiation retinopathy [32-34] or malignancy [32-34] is minimal to none; however, many of these studies had relatively short follow-up times. Although the aforementioned findings seem promising, extended follow-up studies highlight that a subset of patients may develop radiation retinopathy within 10 years of undergoing treatment [35]. Additionally, recent results from the CIRTED post-trial follow up demonstrated that radiotherapy versus sham radiotherapy did not produce any statistical difference in binary clinical composite outcomes such as CAS, total eye score, EUGOGO severity class and ophthalmology index [27]. The study highlighted that radiotherapy did not produce any additional benefits compared with glucocorticoids [27]. Taken together, these findings serve to question the future role of radiotherapy in TED and emphasise the need for further research to determine its efficacy and long-term potential.

Surgical Management

The three main surgical procedures that can be used in the management of TED are orbital decompression, strabismus surgery and eyelid surgery for correction of exophthalmos, diplopia and eyelid retraction, respectively [13]. They are usually carried out in the aforementioned order and patients may not require all surgical procedures [13]. Each of these surgeries has a number of potential complications and long-term sequelae. Complications of orbital decompression include transient facial numbness [36], which can be present in a quarter of patients, corneal erosion, acute subdural haemorrhage, oscillopsia and hematomas [37-41]. A retrospective cohort study involving 448 participants [42] revealed that a quarter of patients undergoing strabismus surgery required re-operations, due to the unpredictable nature of the surgery. Despite the availability of predictive indicators, achieving absolute certainty in predicting the outcome remains a challenge [43,44]. Larger than expected or smaller than expected surgical corrections may be required to correct the deviations [43]. Ultimately, even with the most advanced surgical techniques and well- trained surgeons, surgical complications remain unavoidable. Consequently, given the inevitable challenges and inherent unpredictability of surgical interventions, future research should prioritise on advancing medical treatments for TED.

Biologics

Rituxminab: The use of rituximab has conflicting evidence. An Italian randomised, double-blind, trial supports the use of ritauxmiab [45]. One hundred percent of the participants in the rituximab group vs only 69% of the people in the control group showed CAS improvement in their TED symptoms. More significantly, at one year follow-up assessment, 30.2% of the control group experienced reactivation, whilst no participants in the rituximab group experienced reactivation of their symptoms [45]. However, another randomised, double-masked, placebo-controlled trial conducted concluded that there was no additional benefit of administering ritxaminab over placebo [46]. Furthermore across both studies, rituximab showed no significant improvement in overall quality of life, diplopia or exapthalmos [45,46]. Thus, more long-term robust research is required to validate the use of rituximab in TED.

Tocilizumab: The efficacy of tociluzumab was investigated by a small randomised control trial consisting of 32 patients with corticosteroid resistant TED vs placebo [47]. 93.3% of the participants in the tocilizumab group saw a reduction in CAS by two more points vs 58.8% if the control group [47] at week 16. There was minimal effect on diplopia and propotsis [47]. There were many limitations with this study, mainly its small population, limited follow-up time and long recruitment period. Due to the prolonged recruitment period, it was difficult to ascertain whether some patients had already reached the stable phase of TED during the duration of their treatment [47]. Another more recent observational study of 12 patients concluded that toculizumab was a safe and effective treatment choice [48]. All patients reached disease inactivation by six weeks and achieved a CAS reduction by two points [48]. However, this study again had a limited follow-up period and a small number of patients. More robust large-scale randomised clinical trials, with a longer follow-up time, are warranted to validate these findings.

Teprotumumab: One of the most recent groundbreaking treatment options to be added in the arsenal against TED is teprotumumab. Results from phase 2 clinical trial studies highlighted that teprotumumab [49] compared to placebo at 24 weeks showed higher rates of efficacy (69% vs 20%; P>0.001) [49]. Furthermore, the results highlighted that these effects were rapid. Teprotumumab was able to achieve therapeutic effects in as fast as six weeks compared to placebo (43% vs 4%; P>0.001) [49]. The mean CAS reduction for teprotumumab was able to achieve was a mean decrease in 4 CAS points. The most remarkable finding in this phase 2 clinical trial was that 40% of patients achieved a reduction in proptosis by more than 4mm [49]. Taken together, these results highlight that teprotumumab is effective, induces rapid remission of TED symptoms and also leads to a notable reduction in proptosis, the first for a medical treatment. These findings were supported by results from the phase 3 clinical trial (OPTIC) [50]. Subsequently, data from both trials led to the teprotumumab being approved in the US and Europe [8,13]. The results observed in the aforementioned clinical trials demonstrated efficacy exclusively in active TED. However, recent findings from two large-scale trials [51,52] highlighted similar results when inflammation/disease activity was low. This highlights the potential of teprotumumab to be used in active and inactive disease. Side effects of teprotumumab were noted to be mostly mild; however, reports of muscle spasms, hyperglycemia, alopecia and muscle spasms have been reported [8,13,49-51]. Prior monitoring of different parameters such as blood glucose and audiology testing should be warranted, as well as more ongoing post-market surveillance to minimise risk of side effects as far as possible [13,49-51].

Overall, the results across all studies for teprotumumab mark a revolutionary advancement in medical treatment for TED. The results seen in trials have profound implications and potential clinical advantages compared with current available options. To date, medical therapies have focused on inflammation reduction, and surgical management is utilised to restore the affected eye to its normal anatomical position. Teprotumumab is a first-in-class medication that targets a specific part of the TED pathogenesis pathway. This results in CAS reduction and proptosis improvement in a single effective step, potentially mitigating the need for multiple surgical and medical treatments. This reduces CAS and improves proptosis in a single step, rather than multiple rounds of medical or surgical treatment.

Future management of TED

Subcutaneous and Oral IGF-1R Inhibitors

Following the success that teprotumumab has shown in clinical trials, current research is focusing on streamlining drug delivery systems to achieve maximum therapeutic benefit. Currently, the treatment regime for teprotumumab is a one-hour subcutaneous infusion every three weeks for eight doses [13]. Future versions of teprotumumab are focusing on reformulating and modifying the antibody so it has a longer half-life and can be administered via subcutaneous injections or oral administration [13]. This not only increases the cost effectiveness of teprotumumab but also makes it a more convenient option for patients as they would only need to be given an injection rather than a one-hour infusion [13]. VRDN-001, VRDN-002 and VRDN-003 are three monoclonal antibodies that are currently in different stages of clinical trials; the most promising is potentially VRDN-003 [53,54]. VRDN-003 targets the same location of the IGF-1R as teprotumumab but is modified to be administered for subcutaneous injection with a longer half-life [53]. Preclinical trial data from non-human primates demonstrated remarkable results with VRDN-003, such as doubled half-life, reduced clearance and increased drug concentration in the blood compared to monoclonal antibodies similar to teprotumumab [53]. The dosing regimen of 7.5mg/kg either subcutaneously or intravenously [53]. VRDN-003 has recently also successfully completed phase 1 clinical trials with impressive results. When tested on healthy human participants, VRDN-003 was well tolerated, demonstrated prolonged pharmacodynamics and half-life of around 40-50 days [54]. Taken together, these results highlight the potential for IGF-1R inhibitors to become a convenient, self-administration option for patients. This would provide the patients with greater convenience and autonomy in their treatment, which can in turn serve to improve treatment adherence and quality of life. 

Treating the TSHR K1-70

As previously mentioned, the TSHR is a central component of TED pathogenesis. The TSHR is expressed in retro-orbital fibroblast tissue and also expressed on the thyroid gland itself. A human monoclonal antibody called K1-70 has been designed to target and block the TSHR, therefore preventing the stimulation of the receptor and its subsequent clinical sequelae. Data from preclinical studies conducted on rats and cynomolgus monkeys highlighted that K1-70 was well-tolerated, achieved expected pharmacokinetic parameters and had no off-target effects [55]. In a phase I clinical trial of 18 patients with Grave’s disease and TED, K1-70 was found to be well tolerated and was able to demonstrate clinical improvements in both Graves' disease and TED symptoms [56]. The results are promising and highlight that K1-70 has the potential to simultaneously treat both Graves' disease and TED. The dual-action mechanism of this treatment has the potential to streamline patient care and prevent separate treatment regimes to treat the two conditions. However, despite the promising results, additional research in larger trials is warranted to further validate these findings [56].

IMVT-1401

The IMVT-1401 is a monoclonal antibody that is targeted against the TRAb itself. As previously mentioned, TRAb binds to the TSHR receptor, which results in the subsequent clinical manifestations of TED. A proof-of-concept and clinical trial [57] was conducted on patients with severe to moderate patients with TED [57]. The main outcome measures were a decrease in the serum TRAb level, serum IgG level and proptosis level. The proof-of-concept study highlighted that there was a decrease in TRAb antibodies and the placebo- controlled trial demonstrated a reduction in exophthalmos and CAS responses. However, the proptosis reduction was not significant by week 12 of the study, which indicates that perhaps IMVT-1401 could be more useful if administered earlier in the TED process [57]. It is of note, that the trial was stopped prematurely due to an unexpected increase in low-density lipoprotein cholesterol levels [57]. The exact mechanism of how this occurs is unknown [57]. Whether this increase in cholesterol levels will significantly cause an increase in adverse cardiovascular events also remains unknown [57]. However, preliminary evidence has found that co-administration with a statin could reduce the low-density lipoprotein cholesterol levels [57]. Taken together, whilst IMVT-1401 is promising in reducing the clinical manifestations of TED, further research in clinical trials would be needed to confirm the results and address any unexpected off-target effects.

Small Molecules Targeting TED

Recent research has highlighted that a unique small molecule antagonist can potentially be used to target a specific part of the TSHR and block it. Marcinkowksi and colleges [58] have identified a S37a molecule [58]. This unique small molecule antagonist was discovered by high-throughput screening and relative stereochemistry on hamster cell lines overexpressing the TSHR [58]. The results highlighted that the S37a is highly selective for the TSHR, but also displays a slight positive agnostic effect. This highlights that some low-level activity of the TSHR may remain and it will allow for normal activity of the receptor [58], without the clinical manifestations of TED. The next stage would be to develop the S37a for future pre-clinical and clinical trials. Although this research is in its early stage, S37a development could revolutionise TED treatment, not only it is selective for a specific part TED pathophysiology but it could also be administered orally [59], which would serve to improve ease of administration, especially in needle-phobic patient populations.

## Conclusions

To date, medical treatment focuses on reducing the inflammation in the active stage and surgical management is utilised to correct any post-inflammation distortions of orbital position and function. However, these treatment options are far from perfect; they have variable efficacy and can subject patients to multiple rounds of therapy. Future therapeutic options aim to target specific components of the TED pathway, with the ultimate goal of directly rectifying the clinical manifestations. This is something that has not yet been clinically possible. The next stages would be to develop these therapeutic options in clinical trials and eventually integrate them into clinical practice. If achieved, these treatments have the potential to revolutionise the management of TED.
